# Impact of Depression, Anxiety, and Stress on Mental Health Among Peruvian Healthcare Professionals

**DOI:** 10.3390/healthcare14040490

**Published:** 2026-02-14

**Authors:** Diego Ismael Valencia-Pecho, Silvana Varela-Guevara, Miguel Basauri-Delgado, Jacksaint Saintila

**Affiliations:** 1 Escuela Profesional de Psicología, Facultad de Ciencias de la Salud, Universidad Autónoma del Perú, Lima 15842, Peru; dvalencia@autonoma.edu.pe (D.I.V.-P.); silvana.varela@autonoma.pe (S.V.-G.); mbasauri@autonoma.edu.pe (M.B.-D.); 2 Escuela de Medicina Humana, Facultad de Ciencias de la Salud, Universidad Señor de Sipán, Chiclayo 14001, Peru

**Keywords:** anxiety disorders, depression symptoms, healthcare professionals, mental health, occupational risk factors, psychological distress, Peru

## Abstract

**Background:** Mental health among healthcare professionals is a critical aspect of clinical practice, as they are exposed to demanding working conditions that frequently lead to symptoms of depression, anxiety, and stress. **Objective:** We aimed to examine the influence of depression-, anxiety-, and stress-related symptoms on mental health in healthcare professionals. **Methods:** A quantitative, non-experimental, cross-sectional study was conducted with a sample of 123 healthcare professionals from a Peruvian hospital. Two instruments were administered: the Depression, Anxiety, and Stress Scale (DASS-21) and the Mental Health Inventory–5 (MHI-5). **Results:** More than 75% of healthcare professionals presented mild levels of depression (82.1%) and stress (89.4%), whereas over half reported mild levels of anxiety (63.4%). Significant differences were observed according to age and years of service (*p* < 0.05). Mental health was significantly and inversely correlated with depression (*r* = −0.706), anxiety (*r* = −0.742), and stress (*r* = −0.698). Moreover, the predictive model explained 59.2% of the variance, with a moderate effect size. **Conclusions:** Among healthcare professionals, higher levels of depression, anxiety, and stress were associated with significantly lower mental health. The strength of these correlations highlights the need to better understand and address the negative emotional impact experienced by healthcare personnel.

## 1. Introduction

In recent years, mental health has been severely affected by the COVID-19 pandemic, resulting in various behavioral consequences among the global population [1]. Among the most impacted are frontline workers, such as healthcare personnel, followed by teachers and students, as well as individuals with pre-existing mental disorders [2,3]. The pandemic has had more severe effects in vulnerable contexts where populations are exposed to the disease and lack the necessary resources to cope with the situation [4]. During the first half of 2021, more than 156,000 cases of anxiety, 77,000 cases of depression, and 18,000 cases of psychotropic substance and alcohol use were reported nationwide [5]. Among healthcare professionals specifically, symptoms of anxiety, depression, and stress have increased over time due to the ongoing pandemic context, challenging their coping abilities [6].

Depression is a mood disorder characterized by affective, physical, and cognitive symptoms, with manifestations ranging from sadness and loss of interest in activities to physiological symptoms such as sleep disturbances, fatigue, and difficulty concentrating [7]. This disorder may vary in duration and significantly affects the social, occupational, or academic environment of the individual. In severe cases, it may lead to suicide attempts or suicide [8]. Conversely, anxiety is considered a disproportionate and exaggerated emotional response relative to the actual value of the situation, arising from dysfunctional thinking and distorted evaluation [9]. Stress, meanwhile, is understood as an adaptive response to a specific event. It develops as a natural reaction to new situations; however, if prolonged or intensified over time, it may become a serious phenomenon that affects both mental and physical health. It is therefore associated with threats, fear, and uncertainty, being a very common reaction among healthcare professionals as well as the general population [10].

Regarding previous literature, one study reported an anxiety rate of 23.0%, which was higher among women than men and more prevalent in nurses than physicians [11,12]. Another study conducted in Mexico found that a small percentage of healthcare professionals (physicians, nurses, dentists, and health promoters) exhibited moderate to extremely severe levels of anxiety, as well as mild to moderate levels of depression and stress [13]. In the general population, moderate to severe psychological impact was reported in 53.8% of cases, along with 16.5% presenting depressive symptoms, 28.8% anxiety symptoms, and 8.1% stress, all ranging from moderate to severe [11,12]. Factors associated with high psychological impact and elevated levels of stress, anxiety, and depression included female gender, student status, presence of specific physical symptoms, and negative self-perception of health [14]. Similarly, in Peru, it was found that nursing professionals working in the emergency department of two public hospitals and treating patients suspected of having COVID-19 presented 39.1% anxiety, 24.6% depression, and 8.8% stress. Moreover, characteristics such as age, years of service, and hospital location were identified as being associated with one of these conditions [15].

In the Peruvian context, work in the healthcare sector takes place within an organizational environment characterized by a high care workload, structural limitations, and a strong culture of work commitment, in which overexertion and extended working hours are often normalized, particularly among professionals working in critical care services [16]. This is accompanied by a cultural conception of work centered on responsibility, sacrifice, and a strong sense of vocation, which may hinder the establishment of clear boundaries between work and personal life [17]. In this scenario, work–life balance tends to be compromised, increasing sustained exposure to stress and favoring the emergence of anxiety and depressive symptoms [18]. These organizational and sociocultural characteristics of the Peruvian context may shape how healthcare professionals experience and cope with mental health problems, differentiating their patterns from those observed in other national and international contexts.

Regarding the concept of mental health, it is currently defined as a state of comprehensive well-being that encompasses an individual’s physical, psychological, and social aspects, rather than merely the absence of disease. It refers to the experience of subjective well-being and the ability to function effectively within society [19].

Consequently, a positive psychology perspective and a broader focus on well-being are increasingly being incorporated, becoming key factors in the prevention and intervention of mental disorders. There is growing interest in understanding mental health as a state that promotes positive self-perception, a sense of personal control, and an optimistic outlook on the future and one’s surroundings, which is associated with an optimal level of quality of life [20]. Enjoying good mental health enables individuals to carry out daily activities effectively, cope with stress, and work autonomously and productively. Therefore, mental health becomes a fundamental pillar for achieving individual well-being and ensuring effective performance within the community [19].

Despite the extensive international literature documenting associations between depression, anxiety, stress, and mental health, limited attention has been paid to how these relationships operate within highly demanding clinical environments. In particular, Neonatal Intensive Care Units (NICUs) represent a unique occupational context characterized by sustained emotional exposure, high clinical responsibility, and continuous organizational pressure, which may differentially shape mental health outcomes among healthcare professionals. In Peru, empirical evidence addressing these dynamics within NICU settings remains scarce. Therefore, beyond confirming established associations, the present study seeks to contextualize the relative contribution of depression, anxiety, and stress to overall mental health within a critical care environment, providing applied evidence with potential relevance for workforce monitoring, service organization, and preventive mental health strategies in neonatal care settings.

Therefore, the objective of this study was to examine the association between depression, anxiety, and stress and overall mental health among healthcare professionals working in the Neonatal Intensive Care Unit of a Peruvian social security hospital. Additionally, this study aimed to explore differences in these variables according to sex, age, and years of service, providing context-specific evidence to inform mental health monitoring and intervention strategies in healthcare settings.

A priori, the following general hypothesis was proposed: depression, anxiety, and stress are significantly associated with the mental health of healthcare professionals working in the Neonatal Intensive Care Unit of a Peruvian social security hospital (H1). Additionally, it was hypothesized that significant differences exist in depression, anxiety, stress, and mental health according to sex, age, and years of service (H2).

## 2. Materials and Methods

### 2.1. Study Type and Design

This was a descriptive cross-sectional study.

### 2.2. Participants

The study population consisted of 180 healthcare personnel (physicians, nurses, and nursing technicians), of both sexes, aged between 20 and 65 years (M = 39.6, SD = 6.78), working in the Neonatal Intensive Care Unit of the national hospital Edgardo Rebagliati Martins located in Jesús María. The sample was determined using a statistical formula for finite populations, resulting in 123 healthcare professionals, including 28 men and 95 women. The sampling method was non-probabilistic and purposive (convenience sampling). Inclusion criteria were as follows: being of legal age, working as a healthcare professional, completing the informed consent form, and answering all questionnaire items. Exclusion criteria included: failure to complete informed consent, absence on the day of assessment, and being a health intern or resident. Participation was voluntary, and all participants were informed that they could withdraw from the study at any time without providing a reason and without any negative consequences. Participants were also withdrawn if they chose not to complete the questionnaires or requested the removal of their data.

### 2.3. Instruments Depression, Anxiety, and Stress Scale (DASS-21) [21]

This instrument consists of 21 items and assesses three related negative emotional states: depression, anxiety, and stress. It uses a Likert-type response scale ranging from 0 to 3 points. Severity levels are classified as mild (8–9), moderate (10–12), severe (13–16), and extremely severe (>17). In Peru, Valencia [22] reported adequate fit indices for the three-factor model of the DASS-21 (χ^2^ = 396.80, df = 186, CFI = 0.957, TLI = 0.952, RMSEA = 0.057, SRMR = 0.058). Reliability values for each factor ranged from 0.897 to 0.905.

### 2.4. Mental Health Inventory–5 (MHI-5) [23]

Composed of 5 items that assess mood, psychological well-being, and the absence of distress over the past month through a unidimensional model. It was adapted into Spanish by Rivera-Riquelme et al. [24], who used a Likert-type response scale from 0 to 3 points. The total score ranges from 0 to 15, where higher scores indicate greater psychological well-being and lower scores indicate the opposite. The levels are: low (0–5), medium (6–10), and high (11–15). In Peru, Reyes [25] demonstrated content validity by finding that all items had correlations above 0.20 and were highly significant (*p* < 0.01) with the total score. Convergent validity was established, showing direct correlations between MHI-5 and self-efficacy, subjective happiness, and life satisfaction, and an inverse correlation with depression. Reliability was calculated using Cronbach’s alpha, yielding a value of 0.788, indicating adequate internal consistency.

The sociodemographic variables considered for the comparative analyses were sex (male and female); age (20–30 years, 31–40 years, 41–50 years, and ≥50 years), grouped according to social stages within the adult life cycle, with the last category incorporating older adults due to limited sample size; and years of service (6 months–1 year, 2–3 years, 4–6 years, and ≥7 years), which were distributed into approximately equivalent time periods for comparison purposes.

### 2.5. Procedure

The present study was proposed as a research project to the Vice-Rectorate for Research of the Universidad Autónoma del Perú through a faculty research project call. Once the project was accepted and approved, the corresponding questionnaires were administered.

Before participating in the study, informed consent was requested from all participating physicians and nurses. Detailed information was provided regarding the study objectives, data collection procedures, potential risks and benefits, and assurances of confidentiality and anonymity for information collected via Google Forms. Sociodemographic data were requested, including age, sex, profession, years of service, marital status, monthly salary, and prior COVID-19 diagnosis. The form was distributed through WhatsApp social network between 4 and 29 October 2023.

### 2.6. Statistical Analysis

Once data collection was completed, the information was downloaded into a Microsoft Excel spreadsheet. Subsequently, the data were exported to the statistical software SPSS version 27 in Spanish and Jamovi 2.2.28. These tools enabled descriptive analysis of the variables, including mean (M), standard deviation (SD), as well as the calculation of frequencies and percentages. Additionally, the Kolmogorov–Smirnov goodness-of-fit test with Lilliefors correction was used to confirm the normal distribution of the data. This allowed for the calculation of the Pearson product–moment correlation coefficient, Student’s *t* test for independent samples to assess mean differences, one-way ANOVA, and multiple linear regression, along with their corresponding effect sizes, classified as [26]: small (*d* = 0.20), medium (*d* = 0.50), and large (*d* = 0.80). Prior to conducting the regression analyses, five key statistical assumptions were evaluated. First, the assumption of normality was assessed to examine the distribution of model residuals. Second, the independence of errors was evaluated using the Durbin–Watson statistic. Third, multicollinearity among predictors was examined using variance inflation factor (VIF) values close to unity and tolerance values greater than 0.20. Fourth, the assumption of homoscedasticity was assessed through scatterplots of standardized residuals versus standardized predicted values. Finally, linearity was evaluated using scatterplots to examine the relationship between residuals and predicted values [27,28]. Detailed diagnostic results and graphical outputs are provided in the Supplementary Materials (Tables S1–S4 and Figures S1–S2).

## 3. Results

Table 1 presents the prevalence of depression, anxiety, stress, and mental health levels among the evaluated healthcare professionals. A higher proportion of participants exhibited mild levels of depression (82.1%), anxiety (63.4%), and stress (89.4%). However, problematic levels (severe and extremely severe) accounted for 6.5%, 10.6%, and 2.4%, respectively. Regarding mental health, a greater proportion of participants fell within the high level (47.2%), followed by the medium level (47.2%).

To examine differences in sociodemographic variables related to depression, anxiety, stress, and mental health, Student’s *t* test was used to compare the variable sex, and ANOVA was applied for age and years of service. The data met the assumption of normal distribution (*p* > 0.05). Regarding sex, significant differences were found for mental health, with higher scores reported among women compared to men, with a moderate effect size (*p* < 0.05, *d* = 0.45). However, although anxiety did not show statistically significant differences (*p* > 0.05), it presented a moderate effect size (*d* = 0.43), which was higher in men. With respect to age, significant differences were reported (*p* < 0.05) for all variables analyzed, with a small effect size for depression (ω^2^ = 0.04) and mental health (ω^2^ = 0.05), and a moderate effect size for anxiety (ω^2^ = 0.09) and stress (ω^2^ = 0.06). However, post hoc analyses revealed significant differences only when comparing the 20–30-year group with the ≥51-year group for depression (t = 2.755, *p* = 0.050), anxiety (t = 3.422, *p* = 0.010), stress (t = 3.026, *p* = 0.024), and mental health (t = −3.266, *p* = 0.014). Finally, years of service also showed significant differences across all variables analyzed, with moderate effect sizes (ω^2^ ranging from 0.06 to 0.14) in all cases. In this regard, post hoc analyses identified significant differences in depression (t = 3.194, *p* = 0.013), stress (t = 3.261, *p* = 0.011), and mental health (t = −2.817, *p* = 0.035) when comparing the group with two to three years of service with the group with more than seven years. For anxiety, significant differences were observed between the six months to one year group and the more than seven years group (t = 3.011, *p* = 0.039), as well as between the two to three years group and the more than seven years group (t = 3.359, *p* = 0.008) (see Table 2).

Table 3 presents the Pearson correlations between depression, anxiety, and stress with mental health and its dimensions. Mental health showed moderate and highly significant inverse correlations with depression (r = −0.706; *p* < 0.001), anxiety (r = −0.742; *p* < 0.001), and stress (r = −0.698; *p* < 0.001).

Prior to conducting the multiple linear regression analysis, the assumptions of the regression model were evaluated. First, a normal distribution was observed for the studied variables, with skewness and kurtosis values within the expected range of −2 to +2; additionally, the Shapiro–Wilk test of the residuals confirmed normality (W = 0.984, *p* = 0.168). The assumption of independence of errors was also met, as the Durbin–Watson statistic fell within the acceptable range of 1.5 to 2.5 (2.155). Third, low multicollinearity was identified, given variance inflation factor (VIF) values close to unity and tolerance values greater than 0.20. Moreover, the assumption of homoscedasticity was satisfied, as the scatterplot of standardized residuals versus standardized predicted values showed no evidence of a systematic pattern of increasing or decreasing variance. Finally, linearity between the residuals and fitted values was confirmed. Once these assumptions were verified [27,28], the regression analysis was conducted. For further details, see the Supplementary Materials.

Table 4 presents a multiple linear regression model using the enter method to predict the effect of anxiety, depression, and stress on mental health. The regression equation was statistically significant, *F* (3, 119) = 58.242, *p* < 0.001. The coefficient of determination (*R^2^*) was 0.595, indicating that 59.5% of the variance in mental health scores can be explained by the model’s predictor variables. The model’s effect size was moderate (<0.25) (Domínguez-Lara, 2018) [26]. The regression equation was as follows: Mental health = 12.531 − 0.141 (Anxiety) − 0.294 (Depression) − 0.146 (Stress). This means that mental health scores decrease by 0.141 points for anxiety symptoms, 0.294 points for depressive symptoms, and 0.146 points for stress symptoms. However, depression did not reach statistical significance (*p* > 0.05), which is further confirmed by the confidence intervals of the estimator, as they include zero within the interval.

## 4. Discussion

The present study aimed to determine the influence of depressive, anxiety, and stress symptoms on the mental health of Peruvian healthcare professionals. One of the first findings revealed mild levels of depression (82.1%), anxiety (63.4%), and stress (89.4%), whereas the most prevalent level of mental health was high (48.8%). Similarly, a study conducted by Onofre et al. [13] among healthcare personnel from the Sanitary Jurisdiction VII of Orizaba, Mexico, found that 76.5% showed no anxiety, 80.3% did not meet the criteria for depression, and 82.5% exhibited no signs of stress. However, a small proportion of healthcare professionals present moderate to extremely severe levels of anxiety and depression, as well as mild to moderate stress levels. In contrast, Virto-Concha et al. [29] identified a prevalence of depression (30.8%), anxiety (41.8%), and stress (34.1%) among nursing personnel who were in contact with patients infected with COVID-19 during the health emergency in Cusco, Peru.

These differences in the prevalence and intensity of emotional symptoms can be interpreted in light of the Peruvian organizational and sociocultural context. Within the Peruvian healthcare system, particularly in high-demand services such as intensive care units, a work culture characterized by the normalization of prolonged working hours, high care-related pressure, and a strong orientation toward professional sacrifice and vocational commitment persists [16,17]. These conditions may promote sustained exposure to stress and anxiety, even in the absence of clinically relevant depressive symptomatology, and may affect overall perceptions of mental health [18]. Furthermore, structural limitations and difficulties in balancing work and personal life may help explain why certain emotional symptoms manifest differently compared with other international contexts [16].

When comparing psychological variables by sex among healthcare workers, significant differences (*p* < 0.05) were observed in anxiety and mental health between men and women, with a medium effect size. These findings indicate that men tend to exhibit higher levels of anxiety symptoms, while women report better overall mental health compared to men. For instance, a study conducted in China by Lozano-Vargas [14] also found higher rates of anxiety among female healthcare personnel compared to their male counterparts.

When comparing the studied variables according to the age range of healthcare workers, statistically significant differences were observed in depression, anxiety, stress, and mental health, with effect sizes ranging from small to medium. The age group between 20 and 30 years exhibited higher levels of depressive, anxiety, and stress symptoms compared to other groups. Conversely, mental health—understood as the personal perception of psychological well-being and distress—was higher among participants aged 51 years and older. Similarly, when comparing psychological variables by years of service, statistically significant differences were found in depression, stress, and mental health, with a medium effect size. Healthcare professionals with six months to one year of service showed higher levels of depressive, anxiety, and stress symptoms than those with longer service. On the other hand, participants with more than seven years of service reported better mental health conditions. Likewise, Obando et al. [15] confirmed that emotional variables (anxiety, depression, and stress) differed according to sociodemographic factors such as age, years of service, and hospital location.

Subsequently, the correlational results revealed a moderate and significant inverse relationship between mental health and depression (*r* = −0.706; *p* < 0.001), anxiety (*r* = −0.742; *p* < 0.001), and stress (*r* = −0.698; *p* < 0.001), accompanied by a large effect size [11,12,26]. These findings suggest that as levels of depression, anxiety, and stress decrease, the perception of mental health among healthcare professionals increases. These results are consistent with other studies confirming an inverse relationship between negative emotional symptomatology and mental health [14].

Finally, regarding the regression analysis, it was found that 59.5% of the variance in mental health scores could be explained by the model composed of the predictor variables: depression, anxiety, and stress. In the context of a Neonatal Intensive Care Unit, characterized by sustained emotional demands, high clinical responsibility, and continuous exposure to stressful situations, anxiety and stress may operate as chronic adaptive responses rather than episodic reactions. This context may help explain why these factors show greater explanatory power over perceived mental health, whereas depressive symptoms—often more gradual or internalized—do not emerge as significant predictors. This indicates that higher levels of anxiety and stress are associated with a lower perceived level of mental health. According to the WHO [7,19], maintaining good mental health enables individuals to carry out daily activities while effectively coping with stress, which facilitates autonomous and productive work. Therefore, mental health becomes the cornerstone for achieving individual well-being and effective performance within the community.

### 4.1. Limitations

The present study has several limitations. First, the use of a non-probabilistic sampling method and a relatively small sample size limits the representativeness of the sample and restricts the generalizability of the findings beyond the evaluated institution. Another identified limitation arose from the overlap between indicators of the DASS-21 and MBI-5 instruments, as redundancy among items may generate false positives when correlating the variables; therefore, the results should be interpreted with caution. As such, future studies are recommended with larger sample sizes and probabilistic sampling methods that allow for population-level inferences. Secondly, although this study conducted an explanatory analysis of mental health in relation to anxiety, depression, and stress, it is important to acknowledge the potential mediating or moderating variables that could provide a deeper understanding of this relationship.

### 4.2. Clinical Implications

The findings of this study highlight the importance of systematically addressing mental health among healthcare professionals, particularly in high-demand environments such as hospitals and Neonatal Intensive Care Units (NICUs). Professionals working in NICUs are routinely exposed to high emotional burden, time pressure, ethical dilemmas, and constant vigilance associated with the care of critically ill newborns, which may intensify symptoms of depression, anxiety, and stress and compromise overall mental health. The strong inverse associations observed between these psychological variables and mental health underscore the need for early detection and timely psychological interventions specifically adapted to critical care settings such as NICUs.

From a clinical and organizational perspective, implementing regular mental health screening protocols within NICU teams could facilitate the early identification of professionals at risk and enable timely access to psychological counseling, stress management programs, or occupational mental health services. In NICU settings, where care delivery relies on sustained attention, rapid clinical decision-making, and coordinated teamwork, prioritizing the monitoring of anxiety and stress—rather than focusing exclusively on depressive symptomatology—may represent a more effective strategy for preserving workforce functioning and patient safety. Organizational measures addressing workload distribution, shift scheduling, and protected recovery time should therefore be considered essential components of mental health strategies in neonatal intensive care environments. Psychosocial support strategies—such as mindfulness-based interventions, cognitive-behavioral workshops, and emotional resilience training—should be incorporated into occupational health policies, with particular consideration of the unique emotional and cognitive demands inherent to neonatal intensive care practice.

Furthermore, given the significant differences observed according to sex and age, clinical interventions in NICU contexts should be tailored to relevant sociodemographic characteristics. Younger professionals and those with less clinical experience may benefit from preventive, coping-oriented interventions aimed at strengthening emotional regulation, adaptive coping strategies, and professional support networks within NICU work environments. Finally, integrating structured mental health care into routine institutional practices in NICUs not only supports the psychological well-being of healthcare professionals but may also contribute to safer clinical decision making, improved quality of neonatal care, reduced risk of burnout-related errors, and enhanced staff satisfaction and retention in high-complexity healthcare settings.

## 5. Conclusions

In conclusion, the model confirms that higher levels of anxiety and stress are associated with poorer mental health among healthcare professionals. These results may serve to justify the implementation of psychosocial interventions within healthcare settings. The analysis also reveals significant gender differences in anxiety and mental health, with a moderate effect size, but not in depression or stress. This underscores the importance of considering gender as a relevant factor in the assessment and intervention of mental health within healthcare environments. Furthermore, age significantly influences anxiety, with a moderate effect size, suggesting the need for interventions tailored to different age groups. Although differences were also found in depression, stress, and mental health, their practical relevance is smaller due to the small effect sizes.

## Figures and Tables

**Table 1:** Prevalence of Depression, Anxiety, and Stress, and Mental Health Levels.

Variable	Level	n	%
**Depression**	Mild	101	82.1
	Moderate	14	11.4
	Severe	6	4.9
	Extremely severe	2	1.6
**Anxiety**	Mild	78	63.4
	Moderate	32	26.0
	Severe	6	4.9
	Extremely severe	7	5.7
**Stress**	Mild	110	89.4
	Moderate	10	8.1
	Severe	2	1.6
	Extremely severe	1	0.8
**Mental health**	Low	5	4.1
	Medium	58	47.2
	High	60	48.8

**Table 2:** Differences in Depression, Anxiety, Stress, and Mental Health by Sex, Age, and Years of Service.

Variable	Category	M (SD)	*t*/*F*	*p*	*d*/ω^2^
**Sex**					
**Depression**	Men	4.61 (4.33)	1.03	0.306	0.23
	Women	3.82 (3.29)			
**Anxiety**	Men	5.00 (3.45)	1.97	0.051	0.43
	Women	3.59 (3.29)			
**Stress**	Men	5.57 (3.80)	0.52	0.605	0.11
	Women	5.17 (3.55)			
**Mental health**	Men	9.21 (2.25)	−2.08	0.040	0.45
	Women	10.29 (2.46)			
**Age**					
**Depression**	20–30 years	6.16 (4.36)	3.00	0.033	0.04
	31–40 years	3.62 (3.06)			
	41–50 years	3.76 (4.06)			
	≥51 years	3.14 (1.65)			
**Anxiety**	20–30 years	6.32 (4.44)	5.14	0.002	0.09
	31–40 years	3.86 (2.64)			
	41–50 years	3.30 (3.61)			
	≥51 years	2.29 (2.19)			
**Stress**	20–30 years	7.68 (4.05)	3.86	0.011	0.06
	31–40 years	5.00 (3.14)			
	41–50 years	4.79 (3.95)			
	≥51 years	4.14 (2.65)			
**Mental health**	20–30 years	8.63 (2.08)	3.15	0.028	0.05
	31–40 years	10.12 (2.22)			
	41–50 years	10.33 (2.89)			
	≥51 years	11.00 (2.03)			
**Years of service**					
**Depression**	6 months–1 year	5.79 (5.35)	3.43	0.019	0.07
	2–3 years	5.42 (3.20)			
	4–6 years	4.00 (3.18)			
	≥7 years	3.10 (3.04)			
**Anxiety**	6 months–1 year	6.86 (4.68)	5.21	0.002	0.13
	2–3 years	5.15 (2.85)			
	4–6 years	3.43 (2.82)			
	≥7 years	2.94 (2.88)			
**Stress**	6 months–1 year	6.64 (4.50)	3.68	0.014	0.07
	2–3 years	6.96 (3.40)			
	4–6 years	4.86 (2.98)			
	≥7 years	4.42 (3.34)			
**Mental health**	6 months–1 year	9.00 (2.48)	2.95	0.036	0.06
	2–3 years	9.12 (2.33)			
	4–6 years	9.93 (2.30)			
	≥7 years	10.64 (2.37)			

**Note.** M = mean, SD = standard deviation, *t* = Student’s *t* test, *p* ≤ 0.05, *d* = Cohen’s *d*, F = ANOVA coefficient, ω^2^ = ANOVA effect size.

**Table 3:** Correlations Between Depression, Anxiety, and Stress with Mental Health and Its Dimensions.

Variables	D1: Psychological Well-Being	D2: Psychological Distress	Mental Health
**Depression**	*r* = −0.370 [95% CI: −0.513, −0.206] *p* = 0.000	*r* = 0.759 [95% CI: 0.672, 0.825] *p* = 0.000	*r* = −0.706 [95% CI: −0.784, −0.604] *p* = 0.000
**Anxiety**	*r* = −0.450 [95% CI: −0.580, −0.296] *p* = 0.000	*r* = 0.745 [95% CI: 0.654, 0.814] *p* = 0.000	*r* = −0.742 [95% CI: −0.812, −0.650] *p* = 0.000
**Stress**	*r* = −0.384 [95% CI: −0.525, −0.222] *p* = 0.000	*r* = 0.735 [95% CI: 0.641, 0.807] *p* = 0.000	*r* = −0.698 [95% CI: −0.778, −0.594] *p* = 0.000

**Note.** D1 = Psychological well-being; D2 = Psychological distress; *r* = Pearson’s correlation coefficient; CI = confidence interval; *p* < 0.001.

**Table 4:** Multiple Linear Regression Model for the Effect of Anxiety, Depression, and Stress on Mental Health.

Predictor	*B* [CI 95%]	SE	*t*	*p*	Tolerance	VIF
**Intercept**			49.569			
**Depression**	−0.141 [−0.290, 0.024]	0.080	−3.682	0.094	0.273	3.666
**Anxiety**	−0.294 [−0.449, −0.141]	0.077	−1.827	0.000	0.281	3.558
**Stress**	−0.146 [−0.298, −0.010]	0.072	−2.039	0.039	0.306	3.272

**Note.** *B* = standardized beta coefficient; CI = confidence interval; SE = Standardized error; *t* = Student’s *t* test; *p* = statistical significance; VIF = Variance Inflation Factor.

## Data Availability

The dataset generated and analyzed during the current study is not publicly available due to privacy and ethical restrictions related to the protection of participant anonymity. However, the data may be obtained from the corresponding author upon reasonable request and subject to approval by the research team.

## Supplementary Materials

The following supporting information can be downloaded at: https://www.mdpi.com/article/10.3390/healthcare14040490/s1, Table S1: Item-level descriptive statistics (mean, SD, skewness, kurtosis). Table S2: Regression Model Fit and Independence of Errors. Figure S1: Linearity assessment scatterplot. Table S3: Kolmogorov–Smirnov Normality Test for Study Variables. Figure S2: Scatterplot of Standardized Residuals versus Standardized Predicted Values for the Mental Health Regression Model. Table S4: Multicollinearity Diagnostics (Tolerance and VIF).

## Abbreviations

The following abbreviations are used in this manuscript:

ANOVA	Analysis of Variance
CI	Confidence Interval
COVID-19	Coronavirus Disease 2019
DASS-21	Depression, Anxiety, and Stress Scale–21
D1	Psychological Well-Being (Dimension 1 of MHI-5)
D2	Psychological Distress (Dimension 2 of MHI-5)
df	Degrees of Freedom
M	Mean
MHI-5	Mental Health Inventory–5
OR	Odds Ratio
PAHO	Pan American Health Organization
SD	Standard Deviation
SE	Standard Error
SPSS	Statistical Package for the Social Sciences
SRMR	Standardized Root Mean Square Residual
WHO	World Health Organization
