# Supplementary material for: Impact of Depression, Anxiety, and Stress on Mental Health Among Peruvian Healthcare Professionals
Source: Healthcare (Basel). 2026 Feb 14;14(4):490. doi: 10.3390/healthcare14040490 (PMC12940484; doi:10.3390/healthcare14040490)
Supplement: Supplementary file 1 [file healthcare-14-00490-s001.zip › healthcare-4035129-supplementary.pdf]

SUPPLEMENTARY MATERIAL

I. ITEM DESCRIPTIONS

The descriptive analysis of the items of the variables investigated recognizes the minimum and maximum values, the mean, and the standard deviation. Likewise, asymmetry and kurtosis determine an adequacy within the normal distribution due to values between the interval of -2 to +2.

Supplementary Table S1: Item-level descriptive statistics (mean, SD, skewness, kurtosis)

	Minimum	Maximum	Mean	Std. Deviation	Skewness	Kurtosis
**Depression**	0	18	4.00	3.555	1.267	1.968
**Anxiety**	0	17	3.91	3.373	1.213	1.958
**Stress**	0	17	5.26	3.603	0.443	-0.128
**Mental Health**	2	15	10.05	2.449	-0.561	0.349

II. REGESSION SCENARIOS

1. Linearity: Scatter plot showing the linear relationship between the variables, with a low positive correlation.

Supplementary Figure S1: Linearity assessment scatterplot

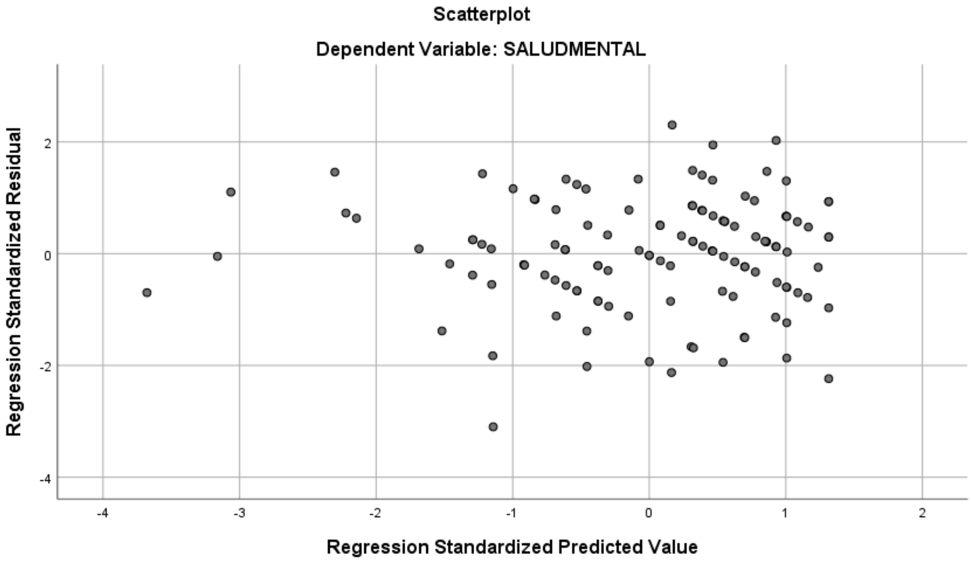

2. Independence of errors: The Durbin–Watson statistic values fall within the range of 1.5 to 2.5, thus satisfying the assumption of independence among the predictors.

Supplementary Table S2. Regression Model Fit and Independence of Errors

Model	R	R Square	Adjusted R Square	Std. Error of the Estimate	Durbin-Watson
1	,771[a]	0.595	0.585	1.578	2.155

Note. [a] Predictors: (Constant), Stress, Anxiety, Depression.

3. Homoscedasticity: The scatterplot of standardized residuals versus standardized predicted values shows a random and relatively constant dispersion, with no evidence of a systematic pattern of increasing or decreasing variance, suggesting that the assumption of homoscedasticity is acceptably met.

Supplementary Figure S2. Scatterplot of Standardized Residuals versus Standardized Predicted Values for the Mental Health Regression Model

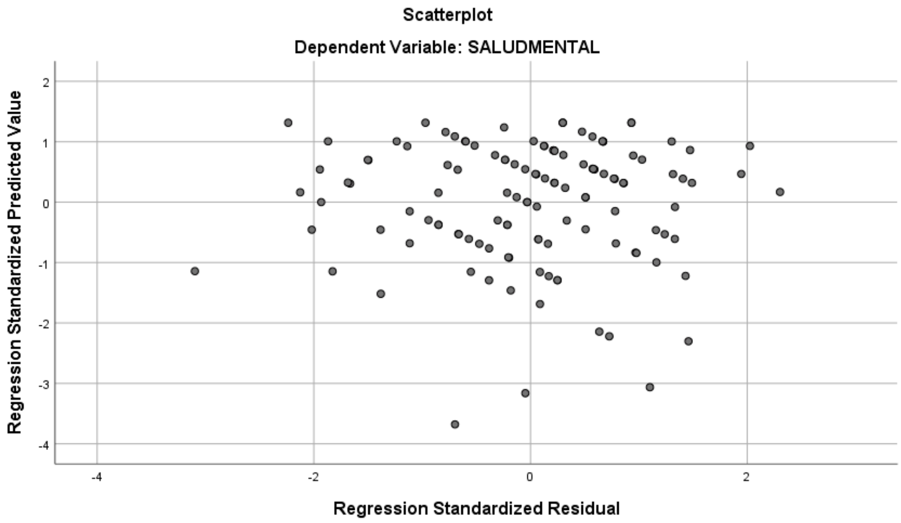

4. Normality: Normal distribution of the variables was observed in the model, as indicated by non-significant results in the normality tests ($p > 0.05$). In addition, normality of the model residuals was confirmed using the Shapiro–Wilk test ($W = 0.984$, $p = 0.168$).

Supplementary Table S3. Kolmogorov–Smirnov Normality Test for Study Variables

Variables	Kolmogorov-Smirnov		
	Statistic	df	Sig.
Depression	0.088	123	0.078
Anxiety	0.052	123	0.210
Stress	0.065	123	0.185
Mental Health	0.087	123	0.081

5. Multicollinearity: Variance inflation factor (VIF) values were below 10 and tolerance values were above 0.20, indicating the absence of problematic multicollinearity among the predictors.

Supplementary Table S4. Multicollinearity Diagnostics (Tolerance and VIF)

Model	Collinearity Statistics	
	Tolerance	VIF
(Constant)		
Depression	0.273	3.666
Anxiety	0.281	3.558
Stress	0.306	3.272
